# Supplementary material for: Linking the rotation of a rigid body to the Schrödinger equation: The quantum tennis racket effect and beyond
Source: Sci Rep. 2017 Jun 21;7:3998. doi: 10.1038/s41598-017-04174-x (PMC5479806; doi:10.1038/s41598-017-04174-x)
Supplement: Supplementary file 1 — Supplementary information [file 41598_2017_4174_MOESM1_ESM.pdf]

Supplementary material of the paper: Linking the rotation of a rigid body to the Schrödinger equation: The quantum tennis racket effect and beyond

L. Van Damme[1], D. Leiner[2], P. Mardešić[3], S. J. Glaser[2], and D. Sugny[1,4*]

[1] *Laboratoire Interdisciplinaire Carnot de Bourgogne (ICB), UMR 6303 CNRS-Université Bourgogne Franche-Comté, 9 Av. A. Savary, BP 47 870, F-21078 DIJON Cedex, FRANCE*

[2] *Department of Chemistry, Technical University of Munich, Lichtenbergstrasse 4, D-85747 Garching, Germany*

[3] *Institut de Mathématiques de Bourgogne, UMR 5584 CNRS-Université de Bourgogne Franche-Comté, 9 Av. A. Savary, BP 47870 21078 Dijon Cedex, France and*

[4] *Institute for Advanced Study, Technische Universität München, Lichtenbergstrasse 2 a, D-85748 Garching, Germany*

(Dated: February 23, 2017)

* dominique.sugny@u-bourgogne.fr

This supplementary material gives a theoretical description of the tennis racket effect in a three-dimensional rigid body and describes the analogy that can be established between the free rotation of a rigid body and the control of the Bloch equations by magnetic fields. This work is organized as follows. Section I summarizes the main results about the two dynamical systems. Two different identifications, corresponding to the cases (a) and (b), are proposed. Sections II and III focus on the analytical derivation of the solutions of the differential systems. We study the robustness of a state to state transfer in the two situations. We also show how the Euler angles can be used to parameterize the global phase of a quantum state. This analysis completes the formal mathematical link between the free rotation of a rigid body and the dynamics of a two-level quantum system. The classical and the quantum tennis racket effects are discussed in Sec. IV. We show in Sec. V how the dynamics of the rigid body can be used to implement one qubit quantum gates. The example of the Hadamard gate is investigated in details. The robustness issue against experimental imperfections of the gates is also discussed. The Montgomery phase, a geometric feature of the free rotation of a rigid body, is derived to realize a non-adiabatic geometric phase gate. A Matlab code computing the trajectories of a rigid body and of the corresponding Bloch vector is also provided and a short description given in Sec. VI. Some standard properties of Jacobi's elliptic functions are detailed in Sec. VII.

I. THE CLASSICAL AND THE QUANTUM DYNAMICAL SYSTEMS

A. A classical rigid body

The free rotation of a rigid body in classical mechanics is based on the motion of its angular momentum $\vec{L}$, which has a constant norm $|\vec{L}| = \ell$ [1, 2]. This norm can be set to $\ell = 1$ without loss of generality [2]. We introduce the frame $(\vec{e}_1, \vec{e}_2, \vec{e}_3)$ attached to the rigid body. These three vectors define the principal axes of inertia of the body. An example is given below with the tennis racket. The time evolution of $\vec{L}$ in the frame $(\vec{e}_1, \vec{e}_2, \vec{e}_3)$ is ruled by Euler's equations:

$$\frac{d\vec{L}}{dt} = \vec{\Omega} \times \vec{L}, \tag{1}$$

where $\vec{\Omega}$ is the angular velocity vector. In matrix form, Eq. (1) reads:

$$\begin{pmatrix} \dot{L}_1 \\ \dot{L}_2 \\ \dot{L}_3 \end{pmatrix} = \begin{pmatrix} 0 & -\Omega_3 & \Omega_2 \\ \Omega_3 & 0 & -\Omega_1 \\ -\Omega_2 & \Omega_1 & 0 \end{pmatrix} \begin{pmatrix} L_1 \\ L_2 \\ L3 \end{pmatrix}. \tag{2}$$

The components of $\vec{\Omega} = (\Omega_1, \Omega_2, \Omega_3)$ in $(\vec{e}_1, \vec{e}_2, \vec{e}_3)$ can be written in terms of the ones of $\vec{L} = (L_1, L_2, L_3)$ as follows:

$$\Omega_1 = \frac{L_1}{I_1}, \quad \Omega_2 = \frac{L_2}{I_2}, \quad \Omega_3 = \frac{L_3}{I_3}, \tag{3}$$

where I_1, I_2 and I_3 are the principal moments of inertia.

For a homogeneous rigid body, the principal moments of inertia are related to the shape of the solid. More precisely, they correspond to the repartition of the mass along the three principal axes of inertia $\vec{e}_1$, $\vec{e}_2$ and $\vec{e}_3$ [2]. For a tennis racket, the principal axes of inertia are such that $\vec{e}_1$ is along the handle, $\vec{e}_2$ is perpendicular to the handle and in the plane defined by the head of the racket, and $\vec{e}_3$ is perpendicular to the head of the racket [8]. In this configuration, we have $I_1 < I_2 < I_3$. The frame attached to the racket is represented in Fig. 1.

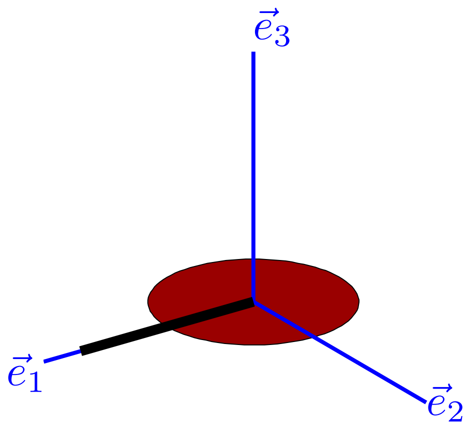

FIG. 1. (Color online) Principal axes of inertia of a tennis racket.

Substituting Eq. (3) into Eq. (1), we can integrate the dynamical system and derive the solutions for $L_1(t)$, $L_2(t)$ and $L_3(t)$. The system has two constants of motion, the total mechanical energy and the angular momentum:

$$\frac{L_1^2}{I_1} + \frac{L_2^2}{I_2} + \frac{L_3^2}{I_3} = 2E, \quad L_1^2 + L_2^2 + L_3^2 = 1.$$

The conservations of the energy and of the angular momentum correspond geometrically to the equation of an ellipsoid of radii $I_1\sqrt{2E}$, $I_2\sqrt{2E}$ and $I_3\sqrt{2E}$, and to a sphere of radius 1, respectively. The classical trajectory $\vec{L}(t)$ lies on the intersection of the two surfaces, and it can be expressed in terms of Jacobi's elliptic functions [2].

B. The Bloch equation

We consider a general two-level quantum system defined by the state $|\lambda(t)\rangle$ whose dynamics is governed by the Schrödinger equation $i\hbar\partial_t|\lambda\rangle = \hat{H}|\lambda\rangle$, where the Hamiltonian $\hat{H}$ is given by:

$$\hat{H} = \frac{\hbar}{2}\begin{pmatrix} \Delta(t) & \Omega(t)e^{-i\eta(t)} \\ \Omega(t)e^{i\eta(t)} & -\Delta(t) \end{pmatrix}. \quad (4)$$

$\Omega(t)$ and $\eta(t)$ are respectively the real amplitude and phase of the control field, and $\Delta(t)$ is the offset with respect to the Larmor frequency of the system [3, 4]. We can first consider the case for which $\eta(t) = 0$. The corresponding Hamiltonian $\hat{H}_A$ reads:

$$\hat{H}_A = \frac{\hbar}{2}\begin{pmatrix} \Delta(t) & \Omega(t) \\ \Omega(t) & -\Delta(t) \end{pmatrix}, \quad (5)$$

A second option consists in working at resonance, setting $\Delta(t) = 0$. The Hamiltonian $\hat{H}_B$ is then given by:

$$\hat{H}_B = \frac{\hbar}{2}\begin{pmatrix} 0 & \omega_1 - i\omega_2 \\ \omega_1 + i\omega_2 & 0 \end{pmatrix}, \quad (6)$$

with $\omega_1 = \Omega\cos\eta$ and $\omega_2 = \Omega\sin\eta$ the two real control fields. The Bloch vector $\vec{M}(t)$ can be defined in terms of the components of the density matrix $\hat{\rho} = |\lambda\rangle\langle\lambda|$ as $M_1 = \hat{\rho}_{21} + \hat{\rho}_{12}$, $M_2 = i(\hat{\rho}_{12} - \hat{\rho}_{21})$, $M_3 = \hat{\rho}_{11} - \hat{\rho}_{22}$, the Bloch equation being derived from the Liouville Von Neumann equation $\dot{\hat{\rho}} = -i/\hbar[H, \hat{\rho}]$. The two choices of parametrization (5) and (6) lead to different Bloch equations:

Case (a) Case (b)

$$\dot{\vec{M}} = \begin{pmatrix} 0 & -\Delta(t) & 0 \\ \Delta(t) & 0 & -\Omega(t) \\ 0 & \Omega(t) & 0 \end{pmatrix}\vec{M};\ \dot{\vec{M}} = \begin{pmatrix} 0 & 0 & \omega_2(t) \\ 0 & 0 & -\omega_1(t) \\ -\omega_2(t) & \omega_1(t) & 0 \end{pmatrix}\vec{M}.$$

It is then straightforward to identify these differential equations with the free rotation of a rigid body given by Eq. (1), with $\vec{M} = \vec{L}$, defining thus some particular families of control fields. Sections II and III will be dedicated to the integration of the Euler equations and to the derivation of the corresponding control fields.

II. INTEGRATION OF THE BLOCH EQUATION: CASE (A)

A. Analytical derivation of the solutions

In case (a), the identification between the two dynamics leads to $\Delta(t) = \Omega_3(t) = M_3(t)/I_3$, $\Omega(t) = \Omega_1(t) = M_1(t)/I_1$ and $\Omega_2(t) = 0$. Note that the condition $\Omega_2(t) = 0$ induces a constraint on the classical rigid body, $I_2 \to \infty$. The two other principal moments of inertia can be set to $I_1 = 1$ and $I_3 = 1/k^2$, $k \in [0, 1]$, without loss of generality. We obtain the following Bloch equation:

$$\dot{M}_1 = -k^2 M_2 M_3, \quad \dot{M}_2 = -(1-k^2) M_1 M_3, \quad \dot{M}_3 = M_1 M_2. \tag{7}$$

The solutions of this system lie in the intersection of the *angular momentum sphere* and of the *energy ellipsoid*. Here, the angular momentum sphere is simply the Bloch sphere, and the energy ellipsoid becomes an elliptic cylinder since $I_2 \to \infty$. More precisely, the equations of the two surfaces are given by:

$$S_1 : \ M_1^2 + M_2^2 + M_3^2 = 1; \quad S_2 : \ M_1^2 + k^2 M_3^2 = 2E.$$

Figure 2 displays the curves corresponding to the intersection of S_1 and S_2 for different energy levels E. Three different families of solutions can be distinguished: the *rotating extremals*, the *oscillating extremals* and the separatrix [2]. The two radii of the elliptic cylinder are $\sqrt{2E}$ and $\sqrt{2E}/k$. The oscillating and rotating extremals are respectively obtained when $\sqrt{2E}/k < 1$ (1 is the radius of the sphere) and $\sqrt{2E}/k > 1$. The separatrix occurs for $\sqrt{2E}/k = 1$. Six equilibrium points belonging to the three axes $\vec{e}_i$ can be found in Fig. 2. The points of the axes $\vec{e}_1$ and $\vec{e}_2$ are stable while the north and the south poles are unstable equilibrium points. The solutions of Eq. (7) are given in Tab. I for the three families of solutions. An important point is that all the trajectories are periodic, except for the separatrix, which connects the two unstable equilibrium points in an infinite time. The control fields are directly related to these trajectories with the identification $\Delta(t) = k^2 M_3(t)$

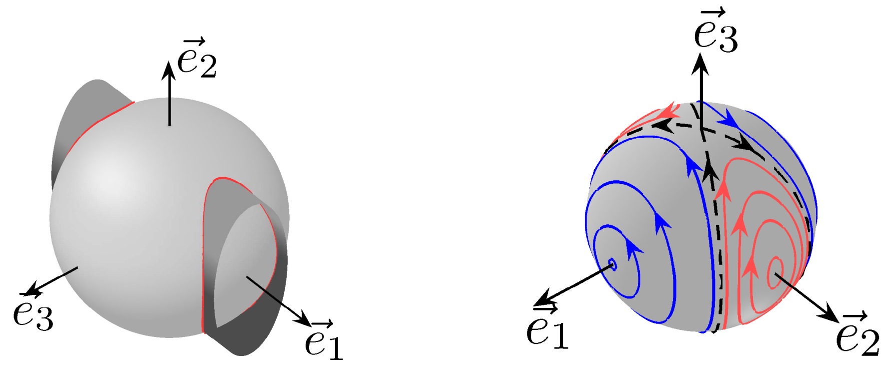

FIG. 2. (Color online) Left panel: Plot of the two surfaces S_1 and S_2 for fixed values of k and E. Right panel: Different trajectories $\vec{M}(t)$ corresponding to different energy levels and a fixed value of k. The red (light gray) curves are the oscillating extremals ($2E < k^2$) and the blue (dark gray) ones are the rotating extremals ($2E > k^2$). The black dashed line depicts the separatrix.

and $\Omega(t) = M_1(t)$. This gives us different families of fields, whose properties depend on the values of the parameters k and E. The control fields are expressed explicitly in Tab. I. The parameter k affects the shape of the elliptic cylinder. For a fixed value of E, k changes the intermediate radius of the ellipsoid, given by $\sqrt{2E}/k$. For a fixed value of k, the parameter E modifies the global size of the cylinder, but not its shape, i.e. the ratio between the radii is preserved.

B. Analysis for a fixed value of k

The rotating extremals define a smooth transition between the solutions associated with the stable point along the vector $\vec{e}_1$ and the separatrix. The stable point occurs when the smallest radius of the elliptic cylinder is equal to the radius of the sphere, that is $2E = 1$. Substituting this value in the rotating field of Tab. I, we get $m = 0$, and then (see Sec. VII for the limits of Jacobi's elliptic functions):

$$\Omega = \frac{S}{\sqrt{1-k^2}}, \quad \Delta = 0, \tag{8}$$

that is a standard constant pulse along $\vec{e}_1$. The separatrix corresponds to the case $2E = k^2$ as shown in Tab. I. This particular pulse given in terms of hyperbolic functions is a general Allen-Eberly solution, which is discussed in Sec. II D. The second stable point occurs in the

Solutions of the Euler equations

Oscillating	Rotating	Separatrix
$2E < k^2$	$2E > k^2$	$2E = k^2$
$M_1 = S\sqrt{2E}\mathrm{cn}(t+\rho,m)$	$M_1 = S\sqrt{2E}\mathrm{dn}(t+\rho,m)$	$M_1 = S_1 k\mathrm{sech}(t+\rho)$
$M_2 = S\frac{\omega}{k}\mathrm{dn}(t+\rho,m)$	$M_2 = S\frac{\omega\sqrt{m}}{k}\mathrm{cn}(t+\rho,m)$	$M_2 = S_2\frac{\omega}{k}\mathrm{sech}(t+\rho)$
$M_3 = \frac{\sqrt{2E}}{k}\mathrm{sn}(t+\rho,m)$	$M_3 = \frac{\sqrt{2mE}}{k}\mathrm{sn}(t+\rho,m)$	$M_3 = S_1S_2\tanh(t+\rho)$
$\omega = k\sqrt{1-2E}$	$\omega = \sqrt{2E(1-k^2)}$	$\omega = k\sqrt{1-k^2}$
$m = \frac{2E(1-k^2)}{\omega^2}$	$m = \frac{k^2(1-2E)}{\omega^2}$	$S_1 = \mathrm{sgn}(M_1(0))$
$S = \mathrm{sgn}(M_2(0))$	$S = \mathrm{sgn}(M_1(0))$	$S_2 = \mathrm{sgn}(M_2(0))$

Control fields of the Bloch equation

Oscillating	Rotating	Separatrix
$\Omega = \frac{S\sqrt{2E}}{k\sqrt{1-2E}}\mathrm{cn}(t+\rho,m)$	$\Omega = \frac{S}{\sqrt{1-k^2}}\mathrm{dn}(t+\rho,m)$	$\Omega = \frac{S_1}{\sqrt{1-k^2}}\mathrm{sech}(t+\rho)$
$\Delta = \frac{\sqrt{2E}}{\sqrt{1-2E}}\mathrm{sn}(t+\rho,m)$	$\Delta = \frac{k\sqrt{m}}{\sqrt{1-k^2}}\mathrm{sn}(t+\rho,m)$	$\Delta = \frac{S_1S_2k}{\sqrt{1-k^2}}\tanh(t+\rho)$

TABLE I. Solutions $\vec{M}(t)$ of the Bloch equation (7) in case (a), with the corresponding control fields. Note that we have $\Omega(t) = M_1(t)/\omega$ and $\Delta(t) = k^2M_3(t)/\omega$. The time is rescaled by a factor ω, in order to simplify the expression of $\vec{M}(t)$. Some properties of Jacobi's elliptic functions $\mathrm{cn}(\cdot,\cdot)$, $\mathrm{dn}(\cdot,\cdot)$ and $\mathrm{sn}(\cdot,\cdot)$ are recalled in Sec. VII. The parameter ρ is a constant phase given by the initial conditions of the dynamics.

oscillating mode, when $2E = 0$. This value leads to a zero field and is not really interesting. The oscillating solutions give a smooth transition between a zero field and an Allen-Eberly type pulse sequence [6].

C. Analysis for a fixed value of E

The parameter k allows us to control the shape of the elliptic cylinder, displayed in Fig. 2, and thus the structure of the trajectories of $\vec{M}(t)$ (see Fig. 3 for an illustration). Note that, for $k = 0$, the rotating solutions are only along $\vec{e}_1$. Substituting $k = 0$ in the rotating fields

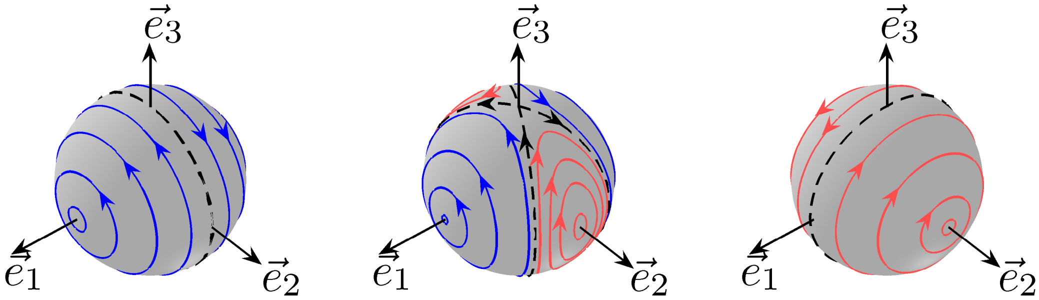

FIG. 3. (Color online) Trajectories $\vec{M}(t)$ on the Bloch sphere for $k \to 0$ (left), $k \in]0,1[$ (middle) and $k \to 1$ (right). The separatrix is associated with some Allen-Eberly type control fields given in Sec. II D.

of Tab. I, we get:

$$\Omega = S, \quad \Delta = 0, \tag{9}$$

which is a constant pulse about $\vec{e}_1$ of amplitude 1. The trajectory along the separatrix is here of the form:

$$\Omega = S_1 \mathrm{sech}(t+\rho), \quad \Delta = 0, \tag{10}$$

with a field area A given by: $A = \int_{-\infty}^{+\infty} |\Omega| dt = \pi$. For $k = 0$, we deduce that the transfer from the north pole to the south pole on the Bloch sphere (called here the inversion of the state) is realized by a π pulse with a hyperbolic secant shape.

For $k = 1$, the rotating solutions do not exist and the different trajectories are parallel to the plane $(\vec{e}_1, \vec{e}_3)$. If we substitute $k = 1$ in the oscillating fields of Tab. I, we obtain:

$$\Omega = \frac{S\sqrt{2E}}{\sqrt{1-2E}} \cos(t+\rho), \quad \Delta = \frac{\sqrt{2E}}{\sqrt{1-2E}} \sin(t+\rho). \tag{11}$$

The components of the Bloch vector can be expressed as:

$$M_1 = S\sqrt{2E}\cos(t+\rho), \quad M_2 = S\sqrt{1-2E}, \quad M_3 = \sqrt{2E}\sin(t+\rho). \tag{12}$$

For a trajectory in the neighborhood of the separatrix, $2E$ is close to 1 and the area of the field Ω goes to infinity. This transfer corresponds to an adiabatic inversion [9].

To summarize, the choice of the value of the parameter k allows us to make a compromise between a constant pulse ($k \to 0$) and an adiabatic pulse ($k \to 1$). Along the separatrix, the solution is an Allen-Eberly solution going from a hyperbolic π pulse for $k = 0$ to an adiabatic inversion when $k \to 1$.

D. The Allen-Eberly solution

The control fields of Tab. I along the separatrix can be viewed as Allen-Eberly type solutions [6, 11–13] of the form:

$$\tilde{\Omega} = \frac{1}{\tau}\sqrt{1+\delta^2\tau^2}\,\mathrm{sech}\left(\frac{\tilde{t}}{\tau}+\rho\right), \quad \tilde{\Delta} = -\delta\tanh\left(\frac{\tilde{t}}{\tau}+\rho\right), \tag{13}$$

where 2δ is the magnitude of the frequency sweep and τ an arbitrary pulse length. In order to simplify the different expressions, we introduce the normalized quantities:

$$\tilde{t} = \frac{t}{\Omega_{ref}}, \quad \tilde{\Omega} = \Omega \times \Omega_{ref}, \quad \tilde{\Delta} = \Delta \times \Omega_{ref}, \quad \Omega_{ref} = \frac{1}{\tau}.$$

We then obtain:

$$\Omega = \sqrt{1+\delta^2\tau^2}\,\mathrm{sech}(t+\rho), \quad \Delta = -\delta\tau\tanh(t+\rho). \tag{14}$$

Using $\delta\tau = k/\sqrt{1-k^2}$, we get the solution on the separatrix (see Tab. I). Starting at $t=0$ on the north pole of the Bloch sphere, i.e. $\rho \to -\infty$, the pulse brings the system to the south pole at $t \to +\infty$. The area A of the pulse is given by:

$$A = \int_0^{+\infty} |\Omega(t)| dt = \frac{\pi}{\sqrt{1-k^2}}.$$

If $k \to 0$, we obtain a standard π pulse which naturally inverts the population of the two-level quantum system. In the case $k > 0$, the area is larger than π, but the transfer is still an inversion. The larger the area is, the more robust the control fields are [9]. Note that Fig. 2 gives an instructive geometrical interpretation of the Allen-Eberly solution. The separatrix connects the north and the south poles of the sphere in an infinite time.

E. Efficiency and robustness of the inversion transfer

In this paragraph, we analyze the properties of three trajectories realizing an inversion process on the Bloch sphere. We define a small positive parameter ϵ corresponding to the initial polar angle of the chosen trajectory. We compare the fields attached to a rotating curve, an oscillating curve and the separatrix as illustrated in Fig. 4. The expression of the fields is given in Tab. I. The phase ρ is computed so that $M_1(0) = 0$ for the oscillating field and $M_2(0) = 0$ for the rotating one, which leads to $\rho = \mathrm{K}(m)$, where K denotes an

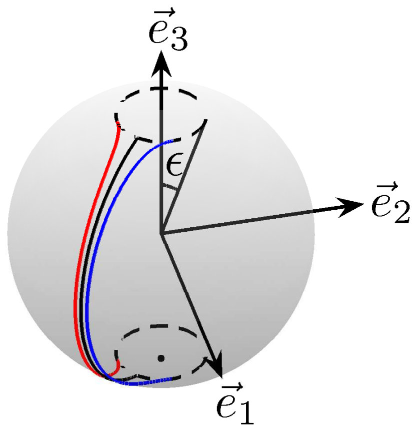

FIG. 4. (Color online) Illustration of the three trajectories selected for the inversion process on the Bloch sphere from the north pole to the south pole. The blue (dark gray), red (light gray) and black curves represent respectively the rotating and oscillating fields and the solution along the separatrix. The parameter ϵ is an arbitrary small angle (see the text for details).

elliptic integral of the first kind [10]. For the separatrix, since $M_3(0) = \cos(\epsilon)$, we can derive ρ as in Tab. II. The time t_f is fixed so that $\vec{M}(t_f)$ is symmetric to $\vec{M}(0)$ with respect to the equatorial plane. The values of all the parameters are given in Tab. II. Table III gives

Oscillating	Rotating	Separatrix
$\Omega = -\frac{\sqrt{2E}}{k\sqrt{1-2E}}\mathrm{cn}(t+\rho,m)$	$\Omega = \frac{1}{\sqrt{1-k^2}}\mathrm{dn}(t+\rho,m)$	$\Omega = \frac{1}{\sqrt{1-k^2}}\mathrm{sech}(t+\rho)$
$\Delta = \frac{\sqrt{2E}}{\sqrt{1-2E}}\mathrm{sn}(t+\rho,m)$	$\Delta = \frac{k\sqrt{m}}{\sqrt{1-k^2}}\mathrm{sn}(t+\rho,m)$	$\Delta = -\frac{1}{\sqrt{1-k^2}}\tanh(t+\rho)$
$2E = k^2\cos^2\epsilon$	$2E = \sin^2\epsilon + k^2\cos^2\epsilon$	$2E = k^2$
$\rho = \mathrm{K}\left(\frac{2E(1-k^2)}{k^2(1-2E)}\right)$	$\rho = \mathrm{K}\left(\frac{k^2(1-2E)}{2E(1-k^2)}\right)$	$\rho = -\mathrm{arctanh}(\cos\epsilon)$
$t_f = 2\rho$	$t_f = 2\rho$	$t_f = 2\vert\rho\vert.$

TABLE II. Expressions of the control fields corresponding to the three curves represented in Fig. 4. The function $K(\cdot)$ denotes the elliptic integral of the first kind.

the final time t_f and the area $A = \int_0^{t_f} |\Omega| dt$ for the three sets of fields. Note that a series expansion around $\varepsilon = 0$ is used to derive simple expressions for t_f and A.

Table III shows that the control time and the area can be changed to some extent by a judicious choice of the parameters ε and k. We observe that, for $k^2 < 1/2$, the rotating field

Oscillating	Rotating	Separatrix
$t_f = 2\ln\left(\frac{4\sqrt{1-k^2}}{\epsilon}\right) + O(\epsilon^2)$ $A = \frac{\pi}{\sqrt{1-k^2}} - \frac{2\epsilon}{1-k^2} + O(\epsilon^3)$	$t_f = 2\ln\left(\frac{4k}{\epsilon}\right) + O(\epsilon^2)$ $A = \frac{\pi}{\sqrt{1-k^2}}$	$t_f = 2\ln\left(\frac{2}{\epsilon}\right) + O(\epsilon^2)$ $A = \frac{\pi}{\sqrt{1-k^2}} - \frac{2\epsilon}{\sqrt{1-k^2}} + O(\epsilon^2).$

TABLE III. Control time and area of the three families of fields for a small value of the parameter ϵ.

allows us to make an inversion in a shorter time t_f, but needs more energy. In contrast, the oscillating mode offers the lowest energy and the shortest time if $k^2 > 3/4$.

Another important feature of the control fields is their robustness property. Here, we focus on the rotating solutions and the parameter ϵ is set to $\epsilon = 10^{-2}$. Figure 5 shows different couples of fields (Ω, Δ) for some values of $k \in [0, 1]$. The robustness is evaluated

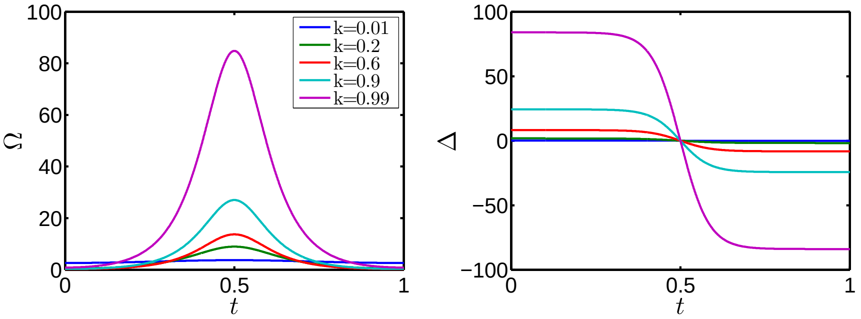

k	0.01	0.2	0.6	0.9	0.99
Area $(\times\pi)$	1.00	1.02	1.25	2.29	7.08

FIG. 5. Control fields computed for $\epsilon = 10^{-2}$ and for different values of k. The time has been normalized to 1 from the scaling: $t' = t/t_f$, $\Omega' = tf \times \Omega$ and $\Delta' = t_f \times \Delta$.

with respect to a scaling factor α on the amplitude of the field and an arbitrary offset term, δ, defined as follows:

$$\Omega^{(\alpha)} = (1+\alpha)\Omega, \quad \Delta^{(\delta)} = \Delta + \delta.$$

We compute the figure of merit $J_3 = -M_3(t_f)$ by propagating the system from $\vec{M}_0 = (0, 0, 1)$ through the Bloch equation of case (a) (see Sec. I). Figure 6 shows the efficiency of the process as a function of the values of δ and α. A better robustness is achieved for larger values of k.

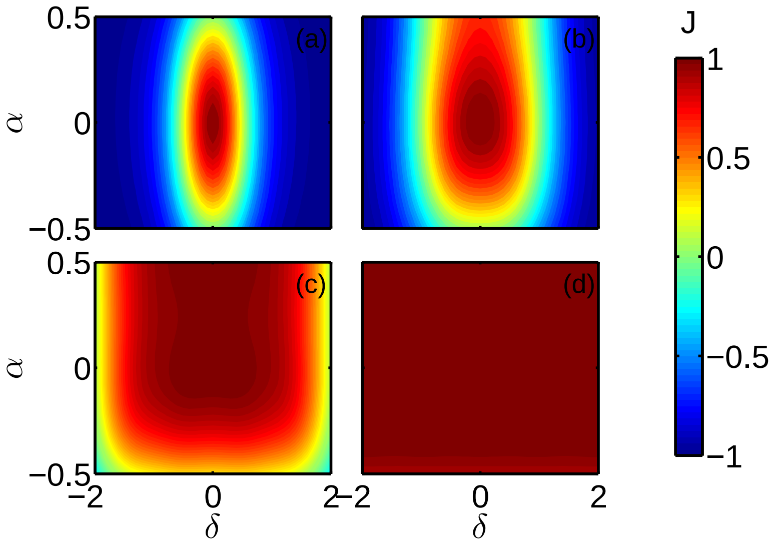

FIG. 6. (Color online) Figure of merit J_3 as a function of the parameters (δ, α). The parameter k is fixed to $k = 0.2$, $k = 0.6$ and $k = 0.9$ and $k = 0.99$ for the panels (a), (b), (c) and (d) respectively.

In summary, the parameter k can be mainly used to adjust the robustness of the fields. The parameter ϵ allows to change the area of the field and the control time.

III. INTEGRATION OF THE BLOCH EQUATION: CASE (B)

A. Analytical derivation of the solution

In case (b) defined in Sec. I, we can identify the Bloch equation with Eq. (1) if $\omega_1(t) = \Omega_1(t) = M_1(t)/I_1$, $\omega_2(t) = \Omega_2(t) = M_2(t)/I_2$, $\Omega_3(t) = 0$, leading to the constraint $I_3 \to \infty$ (we recall that in case (a), $I_2 = +\infty$). We set $I_1 = 1$ and $I_2 = 1/k^2$ and we obtain the differential system:

$$\dot{M}_1 = k^2 M_2 M_3, \quad \dot{M}_2 = -M_1 M_3, \quad \dot{M}_3 = (1-k^2) M_1 M_2. \tag{15}$$

The energy surface is an elliptic cylinder along the vector $\vec{e}_3$:

$$2E = L_1^2 + k^2 L_2^2.$$

The corresponding trajectories are plotted on the Bloch sphere in Fig. 7. We observe that the separatrix makes a transfer on the equator from the state $\vec{M}_0 = (0, 1, 0)$ to $\vec{M}_f = (0, -1, 0)$. This process has some robustness properties, as for the inversion process in case (a). The

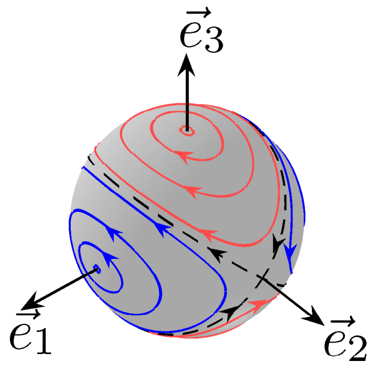

FIG. 7. (Color online) Trajectories $\vec{M}(t)$ plotted on the Bloch sphere for different values of E. The red (light gray) curves are the oscillating extremals ($2E < k^2$) and the blue (dark gray) ones are the rotating extremals ($2E > k^2$). The black dashed line depicts the separatrix, which occurs when $k^2 = 2E$.

corresponding solutions can be derived and are similar to the ones of Tab. I. Only the fields are given in Tab. IV.

Oscillating	Rotating	Separatrix
$\omega_1 = \frac{S\sqrt{2E}}{\omega}\mathrm{cn}(t+\rho,m)$	$\omega_1 = \frac{S\sqrt{2E}}{\omega}\mathrm{dn}(t+\rho,m)$	$\omega_1 = \frac{kS_1}{\omega}\mathrm{sech}(t+\rho)$
$\omega_2 = \frac{k\sqrt{2E}}{\omega}\mathrm{sn}(t+\rho,m)$	$\omega_2 = \frac{k\sqrt{2E}\sqrt{m}}{\omega}\mathrm{sn}(t+\rho,m)$	$\omega_2 = \frac{S_1S_3k^2}{\omega}\tanh(t+\rho)$
$\omega = k\sqrt{1-2E}$	$\omega = \sqrt{2E(1-k^2)}$	$\omega = k\sqrt{1-k^2}$
$m = \frac{2E(1-k^2)}{k^2(1-2E)}$	$m = \frac{k^2(1-2E)}{2E(1-k^2)}$	$S_1 = \mathrm{sgn}(M_1(0))$
$S = -\mathrm{sgn}(M_3(0))$	$S = \mathrm{sgn}(M_1(0))$	$S_3 = \mathrm{sgn}(M_3(0))$

TABLE IV. Control fields associated with the case (b).

B. State to state transfer

We follow here the same approach as the one used in Sec. II E. We consider the transfer from $\vec{M}_0 = (0,1,0)$ to $\vec{M}_f = (0,-1,0)$, through a rotating curve so that:

$$\begin{aligned} M_1(0) &= \sin\epsilon, \quad M_2(0) = \cos\epsilon, \quad\quad M_3(0) = 0 \\ M_1(t_f) &= \sin\epsilon, \quad M_2(t_f) = -\cos\epsilon, \quad M_3(t_f) = 0. \end{aligned} \tag{16}$$

The fields can be expressed as follows:

$$\omega_1 = \frac{1}{\sqrt{1-k^2}}\mathrm{dn}(t+\rho), \quad \omega_2 = \frac{k\sqrt{m}}{\sqrt{1-k^2}}\mathrm{sn}(t+\rho),$$
$$2E = \sin^2\epsilon + k^2\cos^2\epsilon, \quad \rho = \mathrm{K}(m), \quad t_f = 2\rho, \tag{17}$$

with m given in Tab. IV. We set $\epsilon = 10^{-2}$ and we investigate the robustness properties. In this case, the Bloch equation reads:

$$\dot{\vec{M}} = \begin{pmatrix} 0 & -\delta & (1+\alpha)\omega_2 \\ \delta & 0 & -(1+\alpha)\omega_1 \\ -(1+\alpha)\omega_2 & (1+\alpha)\omega_1 & 0 \end{pmatrix} \vec{M}. \tag{18}$$

We consider different control fields for different values of k and we evaluate their robustness as a function of δ and α. The results are shown in Fig. 8.

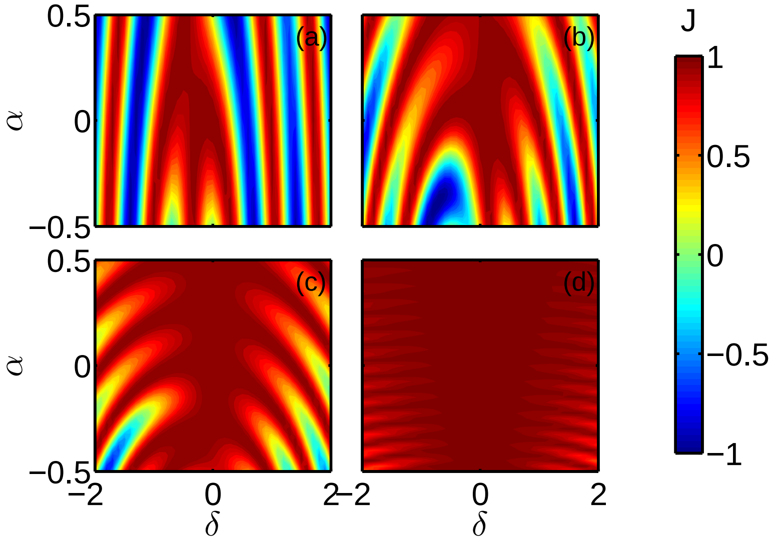

FIG. 8. (Color online) Figure of merit $J_2 = -M_2(t_f)$ as a function of the parameters (δ, α). The parameter k is respectively fixed to $k = 0.2$, $k = 0.7$, $k = 0.9$ and $k = 0.99$ for the panels (a), (b), (c) and (d). In each case, the parameter ϵ is set to 10^{-2}.

C. Global phase

The Bloch vector $\vec{M}(t)$ does not take into account the global phase of the quantum state. We show for the case (b) how to derive this phase by introducing the Euler angles, which completes the analogy between the two-level quantum system and the classical rigid body.

We introduce three Euler angles θ, ϕ and ψ as shown in Fig. 9. The two angles θ and ϕ define the position of the vector $\vec{M}$, and the motion of the frame $(\vec{Q}, \vec{P}, \vec{M})$ with respect to the fixed one $(\vec{e}_1, \vec{e}_2, \vec{e}_3)$ can be described with the third Euler angle ψ.

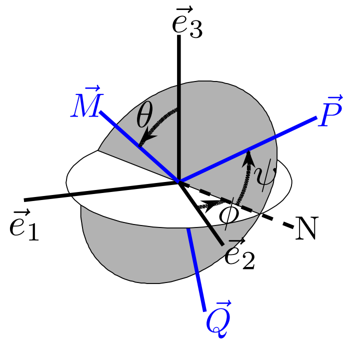

FIG. 9. (Color online) Definition of Euler angles from the frame attached to the Bloch sphere $(\vec{e}_1, \vec{e}_2, \vec{e}_3)$. In a classical system, $(\vec{e}_1, \vec{e}_2, \vec{e}_3)$ is the frame attached to the rigid body. The vector $\vec{M}$ plays the role of the angular momentum $\vec{L}$ of the fictitious rigid body.

The set of angles θ, ϕ and ψ can be used to parameterize the wave function $|\lambda(t)\rangle$ as:

$$|\lambda\rangle = \begin{pmatrix} \cos\left(\frac{\theta}{2}\right) e^{-i\frac{\phi}{2}} \\ \sin\left(\frac{\theta}{2}\right) e^{i\frac{\phi}{2}} \end{pmatrix} e^{-i\frac{\psi}{2}}. \tag{19}$$

The dynamics of the three angles can be obtained by substituting Eq. (19) into the Schrödinger equation (6). We arrive at:

$$\begin{cases} \dot{\theta} = \omega_2 \cos\phi - \omega_1 \sin\phi \\ \dot{\phi} = -\omega_1 \frac{\cos\phi}{\tan\theta} - \omega_2 \frac{\sin\phi}{\tan\theta} \\ \dot{\psi} = \omega_1 \frac{\cos\phi}{\sin\theta} + \omega_2 \frac{\sin\phi}{\sin\theta}. \end{cases} \tag{20}$$

In the Bloch representation, the two angles θ and ϕ define the vector $\vec{M}(t)$ such that:

$$M_1 = \sin\theta\cos\phi, \quad M_2 = \sin\theta\sin\phi, \quad M_3 = \cos\theta. \tag{21}$$

The two control fields can be expressed in terms of the Euler angles as follows:

$$\omega_1 = \frac{1}{\omega}\sin\theta\cos\phi, \quad \omega_2 = \frac{k^2}{\omega}\sin\theta\sin\phi.$$

The final dynamical system to solve can be written as:

$$\begin{cases} \dot{\theta} = -\frac{1-k^2}{\omega}\sin\theta\cos\phi\sin\phi \\ \dot{\phi} = -\frac{1}{\omega}\cos\theta(\cos^2\phi + k^2\sin^2\phi) \\ \dot{\psi} = \frac{1}{\omega}\left(\cos^2\phi + k^2\sin^2\phi\right). \end{cases} \tag{22}$$

D. Rotation matrix

We introduce a 3×3 matrix R defined by $R(t) = (\vec{Q}(t), \vec{P}(t), \vec{M}(t))$, and we denote by R_f the final propagator which satisfies $R(t_f) = R_f R(0)$. The propagator R_f is given by:

$$R_f = R(t_f)^t R(0).$$

The matrix R can be written as a function of the Euler angles, $R = R_\phi R_\theta R_\psi$ with:

$$R_\phi = \begin{pmatrix} \cos\phi & -\sin\phi & 0 \\ \sin\phi & \cos\phi & 0 \\ 0 & 0 & 1 \end{pmatrix},\ R_\theta = \begin{pmatrix} \cos\theta & 0 & \sin\theta \\ 0 & 1 & 0 \\ -\sin\theta & 0 & \cos\theta \end{pmatrix},\ R_\psi = \begin{pmatrix} \cos\psi & -\sin\psi & 0 \\ \sin\psi & \cos\psi & 0 \\ 0 & 0 & 1 \end{pmatrix}. \tag{23}$$

We introduce the angles $(\theta_0, \phi_0, \psi_0)$ and $(\theta_f, \phi_f, \psi_f)$, which are respectively the initial and final values of the Euler angles. The initial global phase ψ_0 is irrelevant, and can be set to 0 without loss of generality.

For a trajectory such that: $\theta_f = \theta_0 = \pi/2$, $\phi_f = -\phi_0$, $\psi_f = (2n+1)\pi$ and $\psi_0 = 0$, the final rotation matrix becomes:

$$R_f = \begin{pmatrix} 1 & 0 & 0 \\ 0 & -1 & 0 \\ 0 & 0 & -1 \end{pmatrix}.$$

Note that this matrix is also the one realized after the application of a π pulse along $\vec{e}_1$. We will see in the next section that for $\psi_f = 3\pi$ $(n = 1)$, the dynamics of this process is associated with the *tennis racket effect* [7]. This effect is described in Sec. IV.

IV. THE CLASSICAL AND THE QUANTUM TENNIS RACKET EFFECTS

The classical tennis racket effect (TRE) is a particular motion which occurs for some trajectories in the neighbourhood of the separatrix [7]. We recall that, for a classical rigid

body, $\vec{e}_1$, $\vec{e}_2$ and $\vec{e}_3$ are the principal axes of inertia of the solid. If the principal moments of inertia are such that $I_1 < I_2 < I_3$ (it is the used convention in this work) then a rotation about the $\vec{e}_1$- and $\vec{e}_3$- axes is stable, but a rotation about $\vec{e}_2$ is unstable. This point can be checked in Fig. 7.

The TRE is easier to figure out by considering the classical case, where $(\vec{X},\vec{Y},\vec{L})$ is the laboratory frame and $(\vec{e}_1,\vec{e}_2,\vec{e}_3)$ is the frame attached to the racket [7, 8]. In this representation, $\vec{e}_1$ is along the handle, $\vec{e}_2$ is perpendicular to the handle and belongs to the head of the racket, and $\vec{e}_3$ is perpendicular to the head. The corresponding process is displayed in Fig. 10.

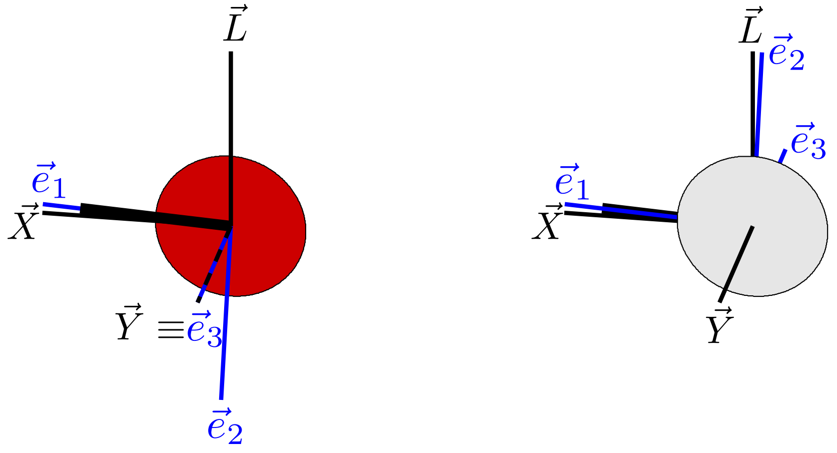

FIG. 10. Illustration of the initial conditions (left), and final conditions (right) of the classical tennis racket effect. The vector $\vec{L}$ is the classical angular momentum of the racket. Note that $\vec{e}_1$ and $\vec{e}_2$ are not exactly collinear to $\vec{X}$ and $\vec{L}$.

For a standard tennis racket, this process occurs for trajectories starting in the neighbourhood of the unstable equilibrium point, i.e. $\vec{L} \simeq \vec{e}_2$ at time $t = 0$. Note that if we apply two times this motion then the racket goes back to its initial position. In other words, if we consider the frame $\mathcal{S} = (\vec{e}_1,\vec{e}_2,\vec{e}_3)$, we have:

$$\mathcal{S}(0) \xrightarrow{1\ \text{TRE}} \begin{pmatrix} 1 & 0 & 0 \\ 0 & -1 & 0 \\ 0 & 0 & -1 \end{pmatrix} \mathcal{S}(0) \xrightarrow{2\ \text{TRE}} \mathcal{S}(0). \tag{24}$$

We have now all the tools in hand to describe the quantum TRE. In the (θ,ϕ,ψ)- represen-

k	0.2	0.4	0.6	0.7	0.9
$\frac{\pi}{2}-\phi_0$	1.55×10^{-7}	4.66×10^{-4}	1.54×10^{-2}	5.18×10^{-2}	0.531

TABLE V. Values of the initial angle ϕ_0 as a function of the parameter k (see the text for details).

tation, the TRE satisfies:

$$\psi_0 = 0, \quad \psi_f = 3\pi, \quad \theta_f = \theta_0 = \pi/2, \quad \phi_f = -\phi_0. \tag{25}$$

If we substitute these relations into the wave function of Eq. (19), we obtain that the wave function goes back to its initial state after the application of four TRE, i.e.

$$|\lambda_0\rangle \overset{\text{1 TRE}}{\longrightarrow} \begin{pmatrix} 0 & -i \\ -i & 0 \end{pmatrix} |\lambda_0\rangle \overset{\text{2 TRE}}{\longrightarrow} \begin{pmatrix} -1 & 0 \\ 0 & -1 \end{pmatrix} |\lambda_0\rangle \overset{\text{3 TRE}}{\longrightarrow} \begin{pmatrix} 0 & i \\ i & 0 \end{pmatrix} |\lambda_0\rangle \overset{\text{4 TRE}}{\longrightarrow} |\zeta_0\rangle.$$

Among all the possible trajectories, only one exactly satisfies the quantum TRE process of Eq. (25). Since $\theta_0 = \pi/2$, we can deduce from Fig. 7 that this curve is a rotating one, starting and ending in the $(\vec{e}_1, \vec{e}_2)$- plane. Unfortunately, the initial conditions of this trajectory are difficult to compute analytically, because the phase ψ can only be expressed in terms of elliptic integrals of the first and third kinds. We thus give directly the numerical values of the coordinates of the initial point for different values of k in Tab. V. Since $\theta_0 = \pi/2$ and $\psi_0 = 0$, only the initial value of ϕ_0 is needed. A TRE is obtained if the trajectory of $\vec{L}(t)$ is close to the separatrix of Fig. 7, which means that $\vec{L}(0)$ starts close to the unstable equilibrium point $(0, 1, 0)$, or equivalently that the initial value of ϕ is of the order of $\pi/2$. The corresponding fields are given by:

$$\begin{aligned} \omega_1 &= \frac{1}{\sqrt{1-k^2}}\mathrm{dn}(t+\rho), \quad \omega_2 = \frac{k\sqrt{m}}{\sqrt{1-k^2}}\mathrm{sn}(t+\rho), \\ 2E &= \cos^2\phi_0 + k^2\sin^2\phi_0, \quad \rho = \mathrm{K}(m), \quad t_f = 2\rho. \end{aligned} \tag{26}$$

We now apply this set of control fields to the quantum system, in order to implement the quantum tennis racket effect. The control process is a unitary transformation which does not depend on the initial conditions. We consider the following initial point:

$$\vec{Q}(0) = \vec{e}_3, \quad \vec{P}(0) = \vec{e}_1, \quad \vec{M}(0) = \vec{e}_2. \tag{27}$$

The TRE-pulse makes a global rotation about the axis $\vec{e}_1$. The three states are given at time t_f by:

$$\vec{Q}(t_f) = -\vec{Q}(0), \quad \vec{P}(t_f) = \vec{P}(0), \quad \vec{M}(t_f) = -\vec{M}(0). \tag{28}$$

We consider the case $k = 0.7$. The control fields are computed from Eq. (26). The dynamics of the vectors $\vec{Q}$, $\vec{P}$ and $\vec{M}$ are plotted in Fig. 11. We also represent in Fig. 11 the motion of the vectors $\vec{e}_1$, $\vec{e}_2$ and $\vec{e}_3$ in the $(\vec{Q}, \vec{P}, \vec{M})$- frame, which is more suitable to describe the classical TRE (in the classical problem, $(\vec{e}_1$,$\vec{e}_2$,$\vec{e}_3)$ is the frame attached to the racket).

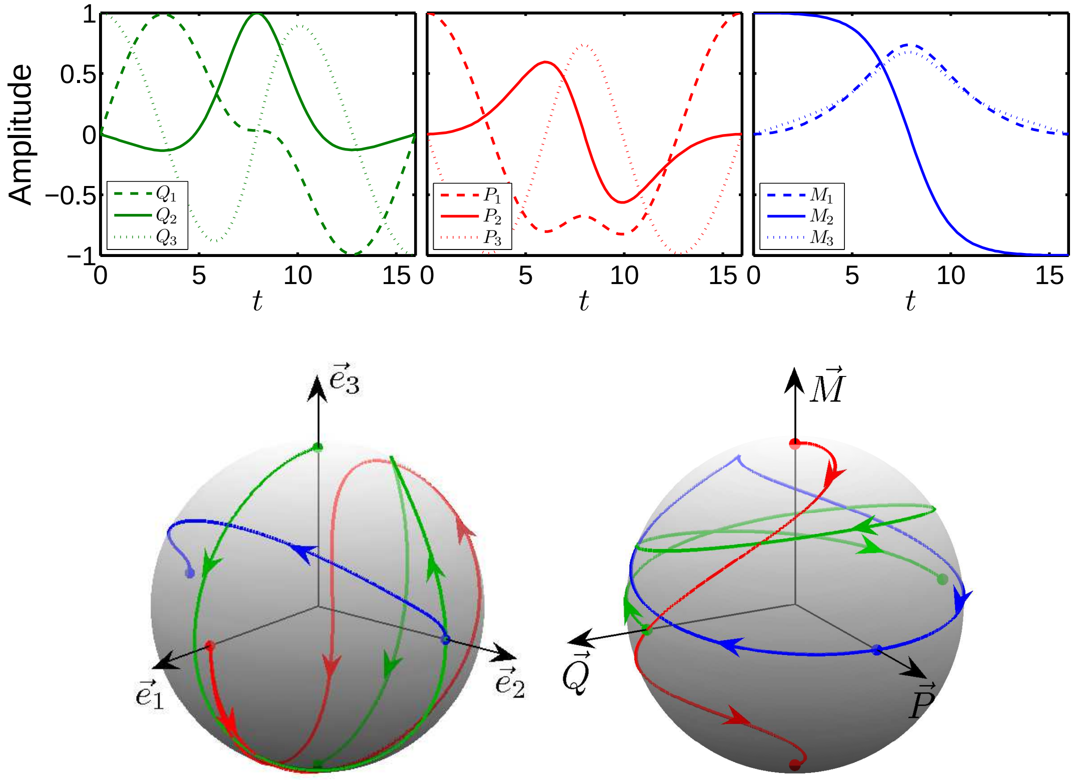

FIG. 11. (Color online) *Upper panels:* Evolution of the coordinates of $\vec{Q}$ (left), $\vec{P}$ (middle) and $\vec{M}$ (right) as a function of time, for $k = 0.7$. The coordinates of the vectors $\vec{M}$, $\vec{P}$ and $\vec{Q}$ are denoted by (M_1, M_2, M_3), (P_1, P_2, P_3) and (Q_1, Q_2, Q_3), respectively. *Lower left panel*: Evolution of $\vec{Q}$ (green- light gray), $\vec{P}$ (red- dark gray) and $\vec{M}$ (blue- black) on the Bloch sphere. *Lower right panel:* Motion of $\vec{e}_1$ (blue- black), $\vec{e}_2$ (red- dark gray) and $\vec{e}_3$ (green- light gray) in the frame $(\vec{Q}, \vec{P}, \vec{M})$. This dynamics corresponds to the one of the quantum tennis racket effect, where the dynamics of $\vec{M}$ is similar to the dynamics of the classical angular momentum.

V. IMPLEMENTATION OF QUANTUM GATES

The goal of this section is to show how the dynamics of a rigid body can be used to implement one-qubit quantum gates. In Sec. V A, we consider the example of the Hadamard

gate before generalizing this result to any one-qubit quantum gate in Sec. V B. Section V C is dedicated to the case of geometric phase gates. The robustness issue of the gates with respect to experimental imperfections is investigated in Sec. V D. Generalizing the BIR approach used in NMR [27, 28], we show that the quantum gates can be made robust. As an illustrative example, we consider the case of a NOT gate and its robustness against control field inhomogeneities.

A. The Hadamard gate

The Hadamard gate U_H is a unitary transformation which can be decomposed into a π phase gate and a $\frac{\pi}{2}$ rotation gate. It reads:

$$U_H = -\frac{i}{\sqrt{2}} \begin{pmatrix} 1 & 1 \\ 1 & -1 \end{pmatrix}. \tag{29}$$

We can show that this gate corresponds to a transformation G_H in $SO(3)$:

$$G_H = \begin{pmatrix} 0 & 0 & 1 \\ 0 & -1 & 0 \\ 1 & 0 & 0 \end{pmatrix}. \tag{30}$$

In a classical rigid body, the Hadamard gate realizes the following transfer:

$$R(0) \to R(t_f) = G_H R(0), \tag{31}$$

where R is the rotation matrix given in terms of Euler's angles. Since the classical angular momentum $\vec{L}$ must also satisfy the transfer (31), we deduce that:

$$L_1(t_f) = L_3(0), \quad L_2(t_f) = -L_2(0), \quad L_3(t_f) = L_1(0). \tag{32}$$

We choose a trajectory such that $L_1(0) = L_3(0)$ and $L_1(t_f) = L_3(t_f)$ at time t_f, which ensures that the Hadamard transformation is satisfied for $\vec{L}$, due to the symmetries of the Jacobi functions. These conditions can be verified for a rotating extremal if $k < 1/\sqrt{2}$, an oscillating one if $k > 1/\sqrt{2}$ and along the separatrix if $k = 1/\sqrt{2}$. As an illustrative example, we set $k = 0.5$. The solution is a rotating extremal of the form:

$$\begin{cases} L_1(t) = \sqrt{2E}\, \mathrm{dn}(t+\rho, m), \\ L_2(t) = -\frac{\sqrt{2Em}}{k}\, \mathrm{sn}(t+\rho, m), \\ L_3(t) = \sqrt{1-2E}\, \mathrm{cn}(t+\rho, m). \end{cases} \tag{33}$$

It can be shown that the initial condition $L_1(0) = L_3(0)$ implies that the parameter ρ is given by:

$$\rho = -\mathrm{F}\left[\sqrt{\frac{m(1-k^2)-k^2}{m(1-2k^2)}}, m\right]. \tag{34}$$

The condition at time t_f is satisfied if we choose t_f such that:

$$t_f = 2|\rho|. \tag{35}$$

Equations (34) and (35) ensure that $\vec{L}$ satisfies the conditions of the Hadamard gate. However, another relation is required on the global phase of the state. It is given by the final value of the euler angle $\psi(t_f)$ (we recall that at time $t = 0$, we can set $\psi(0) = 0$). Using the relations

$$L_1 = \sin\theta\cos\phi, \quad L_2 = \sin\theta\sin\phi, \quad L_3 = \cos\theta, \tag{36}$$

we obtain that $\theta(t_f) = \theta(0)$ and $\phi(t_f) = -\phi(0)$. Moreover, since the rotation matrix R can be written as $R = R_\phi R_\theta R_\psi$, where R_ϕ, R_θ and R_ψ are defined in Eq. (23), straightforward computations lead to the following global phase $\psi(t_f)$:

$$\tan\psi(t_f) = \sqrt{\left(\frac{L_2(0)}{L_1(0)}\right)^4 + 2\left(\frac{L_2(0)}{L_1(0)}\right)^2}. \tag{37}$$

The Hadamard gate is associated with a trajectory of the rigid body satisfying Eqs. (34), (35) and (37). However, the global phase ψ can only be expressed in terms of elliptic integrals of the third kind. A numerical solution for $k = 0.5$ is given by the following initial angular momentum:

$$L_1(0) = 0.1331, \quad L_2(0) = 0.9821, \quad L_3(0) = L_1(0). \tag{38}$$

The corresponding trajectories of the frames $(\vec{X}, \vec{Y}, \vec{L})$ and $(\vec{Q}, \vec{P}, \vec{M})$ of the classical and quantum systems are displayed in Fig. 12.

B. Generalization to any one-qubit gate

The goal of this paragraph is to show that the trajectories of the free rotation of a rigid body allow us to realize any one-qubit gate. We first present a proof showing that the reachable set of the control protocol is SO(3) and then a numerical method that can be applied for any gate.

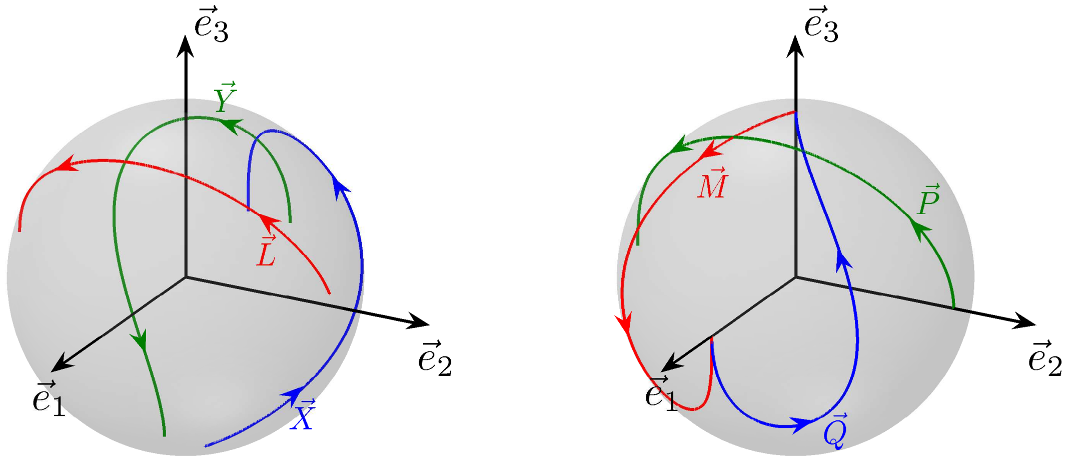

FIG. 12. (Color online) *Left panel:* Hadamard gate in the classical system. *Right panel:* Hadamard gate in the quantum system. In this case, at time $t = 0$, we have $\vec{Q}(0) \equiv \vec{e}_1$, $\vec{P}(0) \equiv \vec{e}_2$, $\vec{M}(0) \equiv \vec{e}_3$.

The rotation matrix $R(t)$ associated with the classical system can be viewed as the optimal solution of the following dynamical system:

$$\dot{R} = \begin{pmatrix} 0 & 0 & ku_2 \\ 0 & 0 & -u_1 \\ -ku_2 & u_1 & 0 \end{pmatrix} R, \tag{39}$$

with the constraint $u_1^2 + u_2^2 = 1$. Note that this system is fully controllable, which means that any rotation matrix R can be realized.

We denote by a_{ij} the element of the ith row and jth column of the matrix R. We introduce $\vec{r}$ the vector defined as $\vec{r} = {}^t(a_{11}, a_{21}, \cdots, a_{33})$, and we define the energy minimum pseudo-Hamiltonian H_p of the Pontryagin Maximum Principle (See Ref. [14] for mathematical details):

$$H_p = \vec{p} \cdot \dot{\vec{r}} - \tfrac{1}{2}\left(u_1^2 + u_2^2 \right), \tag{40}$$

where $\vec{p} = {}^t(b_{11}, b_{21}, \cdots, b_{33})$ is the adjoint state vector. We define the following angular momentum:

$$\vec{L} = \begin{pmatrix} L_1 \\ L_2 \\ L_3 \end{pmatrix} = \sum_{n=1}^{3} \begin{pmatrix} b_{1n} \\ b_{2n} \\ b_{3n} \end{pmatrix} \times \begin{pmatrix} a_{1n} \\ a_{2n} \\ a_{3n} \end{pmatrix}. \tag{41}$$

The pseudo-Hamiltonian can be written in terms of the component of this angular momen-

tum as:

$$H_p = u_1 L_1 + k u_2 L_2 - \tfrac{1}{2}\left(u_1^2 + u_2^2 \right). \tag{42}$$

The Pontryagin Maximum Principle states that the dynamics and the associated control fields are optimal if this pseudo-Hamiltonian is maximum. The Pontryagin Hamiltonian $\mathcal{H}$ is defined so that $\mathcal{H} = \max_{u_1^2+u_2^2=1} H_p$. This optimality condition is satisfied for the following control fields $u_1 = \frac{L_1}{\sqrt{L_1^2+k^2L_2^2}}$ and $u_2 = \frac{kL_2}{\sqrt{L_1^2+k^2L_2^2}}$. In this case, we get that $2E = L_1^2 + k^2 L_2^2$ is constant and we can show that the dynamics of the angular momentum $\vec{L}$ satisfies the Euler's equation (15).

In order to illustrate the fact that any gate can be realized, we propose a numerical method to obtain the following gate:

$$R_f = \frac{1}{4}\begin{pmatrix} -\frac{\sqrt{6}}{2} & -\frac{3\sqrt{6}}{2} & 1 \\ \frac{5\sqrt{2}}{2} & -\frac{\sqrt{2}}{2} & \sqrt{3} \\ -\sqrt{2} & \sqrt{2} & 2\sqrt{3} \end{pmatrix}. \tag{43}$$

We define the function $F = \frac{1}{3}\text{Trace}[{}^tR_f \cdot (R(t) \cdot {}^tR(0))]$, where $R(t)$ is the rotation matrix associated with the classical system. We recall that the product $R(t) \cdot {}^tR(0)$ gives the value of the gate realized by the classical system at time t. We fix a maximum value for the time $T_{\max} = 4\text{K}(m)$ which corresponds to a complete revolution of $\vec{L}(t)$, and we integrate the system (22) numerically from $t = 0$ to $t = T_{\max}$ for different initial points $(\theta(0), \phi(0))$. We determine numerically the value F^* for which F is maximum, and the corresponding time T^*. We then plot F^* as a function of $\theta(0)$ and $\phi(0)$. The result is displayed in Fig. 13 for $k = 0.5$. This analysis allows us to determine the initial point $(\theta(0), \phi(0))$ and the final time $t_f = T^*$ of the gate. We obtain:

$$\theta(0) = 0.7104, \quad \phi(0) = -0.6078, \quad t_f = 6.543, \tag{44}$$

for $k = 0.5$.

C. Geometric phase gate

The free rotation of a rigid body has a geometric feature called the Montgomery phase [15, 16]. This phase can be defined by considering one period of the angular momentum $\vec{M}$ in the frame $(\vec{e}_1, \vec{e}_2, \vec{e}_3)$. During this motion, the frame $(\vec{Q}, \vec{P}, \vec{M})$ rotates about $\vec{M}$ by an angle

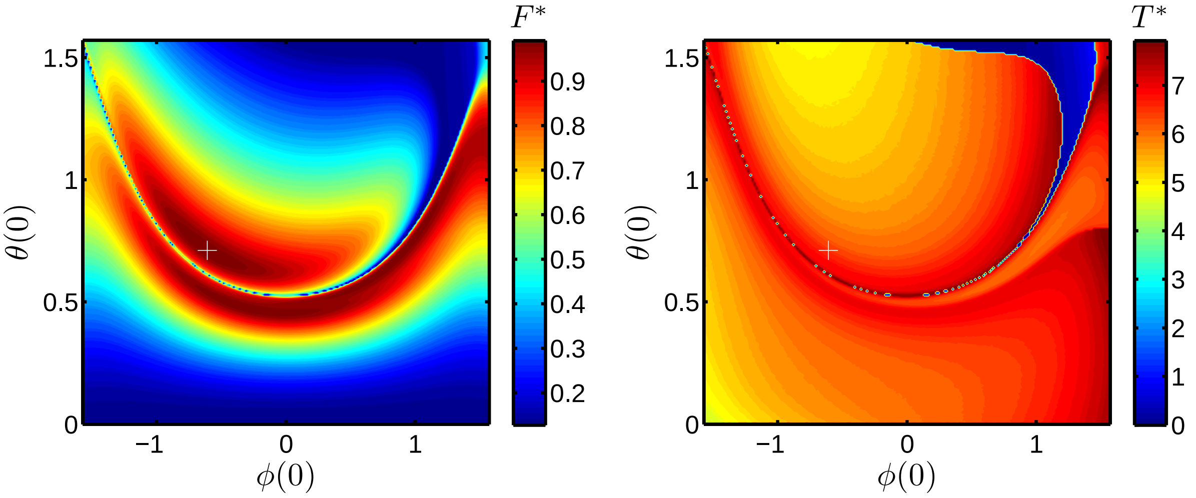

FIG. 13. (Color online) Evolution of F^* as a function of $(\theta(0), \phi(0))$ (left), and of the corresponding time T^* (right) for $k = 0.5$. The cross depicts the point $(\theta(0), \phi(0))$ for which the gate is realized with a precision of $1 - F^* = 1.69 \times 10^{-5}$. Note that this solution is not unique. We choose the one with the shortest time transfer.

$\Delta\psi$ (see Fig. 9), which is the Montgomery phase. This phase can be expressed as the sum of a dynamical and a geometric parts as follows:

$$\Delta\psi = \frac{2ET}{M} - S,$$

where M is the norm of the angular momentum, T the period of the motion of $\vec{M}$ and S is the solid angle swept out by the angular momentum vector in the frame $(\vec{e}_1, \vec{e}_2, \vec{e}_3)$. Starting from the conservation of the energy $2E = M_1^2 + k^2 M_2^2$, and using Eq. (22) we have:

$$2E = \sin^2\theta(\cos^2\phi + k^2\sin^2\phi) = \omega(1 - \cos^2\theta)\dot{\psi}. \tag{45}$$

From $\cos^2\theta\dot{\psi} = -\cos\theta\dot{\phi}$, we get:

$$d\psi = \frac{2E}{\omega}dt - \cos\theta d\phi = \frac{2E}{\omega}dt - \left(\int_{\pi/2}^{\theta} \sin\theta' d\theta'\right) d\phi. \tag{46}$$

Thus, the variation $\Delta\psi$ of the phase for one period is given by:

$$\Delta\psi = \frac{2ET}{\omega} - S, \tag{47}$$

where $S = \int_{\phi_0}^{\phi_f} \int_{\pi/2}^{\theta(\phi)} \sin\theta d\theta d\phi$ is the solid angle swept out by $\vec{M}$. This formula is of the same form as the well-known Berry phase [17, 18] in quantum mechanics which can be used for

adiabatic [18] and periodic [19] trajectories on the Bloch sphere. Such geometric phases have been recently the subject of a large interest in quantum computing as a way to implement geometric and thus robust quantum gates [20, 21]. One of the main difficulties to implement phase gates using geometric phases is to find a way to cancel the dynamical contribution of the phase. Different techniques have been proposed up to date [20, 21]. For instance, Jones et al. [22] showed that the dynamical part of Berry's phase can be removed by using two cyclic trajectories of $\vec{M}(t)$ on the Bloch sphere, the second cycle being surrounded by a pair of π pulses. In [22], the geometric phase gate was implemented in the adiabatic regime, but it is possible to generalize this process to consider non-adiabatic cyclic evolution [23–26].

We propose here a method based on the free rotation of a rigid body and the TRE to implement a geometric phase gate in the non-adiabatic regime. We first recall that each trajectory $\vec{M}(t)$ of Fig. 7 can evolve in the backward direction by changing the sign of the control field $\omega_1(t)$ (see Sec. I). If we consider two identical cycles, the second being followed in the backward direction along the same trajectory, then all the phases vanish, the dynamical ones as well as the geometric ones. However, if for the second cycle, the value of k is different from the first cycle, the total phase at the end of the process is given by $\Delta\psi_{cycle\,1} - \Delta\psi_{cycle\,2}$. This process does not cancel automatically the dynamical phases. To do so, a particular trajectory for the second cycle has to be chosen.

The method can be described as follows. To simplify the discussion and the analytical computations, we assume here that the system follows trajectories along the separatrices. A similar process can be designed by considering trajectories associated with the TRE, which are close to the separatrices. More precisely, we first make a transfer along the separatrix from the point $(0, 1, 0)$ to the point $(0, -1, 0)$ with a finite (long enough) time, and a fixed value k_a. The second step consists in bringing the system from $(0, -1, 0)$ to $(0, 1, 0)$ also along the separatrix but with a different value of the parameter k, i.e. $k_b \neq k_a$. Since the time T_b of the second process can be chosen arbitrarily long, we choose T_b such that the two dynamical phases cancel each other. The global process is shown in Fig. 14 and described in Tab. VI.

In order to derive the geometric phase, we consider the case of a separatrix such that $M_1(t) > 0$ and $M_3(t) > 0$ for $t > 0$. We start from the following expression of the geometric

phase:

$$S = \int_{-\pi/2}^{\pi/2} \int_{\pi/2}^{\theta(\phi)} \sin\theta d\theta d\phi = -\int_{-\pi/2}^{\pi/2} \cos\theta(\phi) d\phi. \tag{48}$$

For this separatrix, we have $\theta \in [0, \pi/2]$, which gives $\cos\theta = \sqrt{1-\sin^2\theta}$. Along the separatrix, $2E = k^2$ and we can express $\sin^2\theta$ in terms of the angle ϕ using Eq. (45). We deduce that:

$$S = -\int_{-\pi/2}^{\pi/2} \sqrt{\frac{1-k^2-(1-k^2)\sin^2\phi}{1-(1-k^2)\sin^2\phi}} = -\int_{-\pi/2}^{\pi/2} \sqrt{1-k^2} \frac{\cos\phi}{\sqrt{\cos^2\phi + k^2\sin^2\phi}}. \tag{49}$$

Finally, the integration gives:

$$S = -2\arcsin(\sqrt{1-k^2}). \tag{50}$$

We recall that the system evolves in the forward and backward directions for the cycles (a) and (b), respectively. Moreover, on the separatrix we have $\omega = k\sqrt{1-k^2}$ and $2E = k^2$. Thus, for each cycle, the variation $\Delta\psi$ is given by:

$$\Delta\psi_a = \frac{k_a T_a}{\sqrt{1-k_a^2}} + 2\arcsin(\sqrt{1-k_a^2}), \quad \Delta\psi_b = -\frac{k_b T_b}{\sqrt{1-k_b^2}} - 2\arcsin(\sqrt{1-k_b^2}). \tag{51}$$

If we choose T_b such that:

$$T_b = \frac{k_a\sqrt{1-k_b^2}}{k_b\sqrt{1-k_a^2}} T_a, \tag{52}$$

then the two dynamical phases cancel each other and we get a purely geometric phase given by:

$$\Delta\psi_{tot} = \Delta\psi_a + \Delta\psi_b = 2[\arcsin(\sqrt{1-k_a^2}) - \arcsin(\sqrt{1-k_b^2})]. \tag{53}$$

Going back to the expression of the quantum state of Eq. (19), this corresponds to a phase gate of the form

$$\begin{pmatrix} 1 & 0 \\ 0 & e^{i\Delta\psi_{tot}} \end{pmatrix}. \tag{54}$$

The global process with the applied control fields is described in Tab. VI. The final geometric phase is equal to the area displayed in Fig. 14. In Fig. 14, we consider the following parameters $T_a = 100$, $k_a = (\sqrt{2-\sqrt{2}})/2$ and $k_b = (\sqrt{2+\sqrt{2}})/2$ in order to implement a geometric phase equal to $\Delta\psi_{tot} = \pi/2$.

Path a	Path b
$t \in [0, T_a]$	$t \in [T_a, T_a + T_b]$
T_a long enough	$T_b = \frac{k_a\sqrt{1-k_b^2}}{k_b\sqrt{1-k_a^2}} T_a$
$\omega_1(t) = \frac{1}{\sqrt{1-k_a^2}}\mathrm{sech}(t + \rho_a)$	$\omega_1(t) = -\frac{1}{\sqrt{1-k_b^2}}\mathrm{sech}(t + \rho_b)$
$\omega_2(t) = -\frac{1}{k_a\sqrt{1-k_a^2}} \tanh(t + \rho_a)$	$\omega_2(t) = -\frac{1}{k_b\sqrt{1-k_b^2}} \tanh(t + \rho_b)$
$\rho_a = -T_a/2$	$\rho_b = -T_b/2 - T_a$.

TABLE VI. Description of the process for implementing a phase gate. Note that the sign of ω_1 changes in the cycle (b) in order to follow the second trajectory in the backward direction.

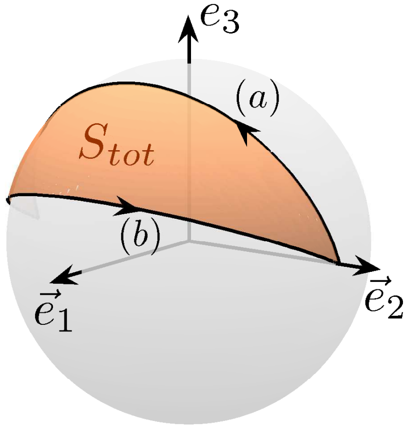

FIG. 14. (Color online) Trajectories on the Bloch sphere for implementing a $\pi/2$ phase gate. The brown (gray) surface is the total geometric phase at the end of the process i.e. $S_{tot} = \pi/2$.

D. Robustness of the NOT gate

We show in this paragraph how the robustness of the control fields used to implement one-qubit gates can be improved. The approach is inspired from the BIR- pulses method in NMR [27, 28]. We design a pulse which allows us to realize a NOT gate in a robust manner with respect to the control fields- inhomogeneities, which is equivalent to a BIR-1 pulse in the adiabatic limit [27, 28]. We use the fact that the global phase of the state monotonically increases or decreases when the system follows a specific trajectory of the phase portrait. This property allows us to cancel the global phase of the dynamics (both dynamical and geometrical terms) and then to realize a robust NOT gate. As an example, we consider here the separatrix of the configuration (a) (see Fig. 2).

The separatrix is first followed from the north pole to the equatorial plane of the sphere in the direction given in Fig. 2. We then transfer the state to another separatrix followed in the opposite direction in order to reach the south pole of the Bloch sphere. The two separatrices are connected with a constant pulse $\Delta(t)$ about $\vec{e}_3$. The global phase is cancelled leading to an inversion with a phase equal to zero, i.e. a NOT- gate along the $\vec{e}_2$- axis. The trajectory is represented in Fig. 15. We can show that the azimuthal angle between the two separatrices

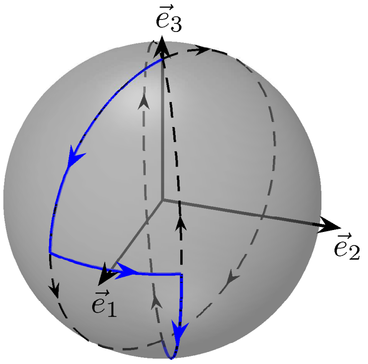

FIG. 15. (Color online) Trajectory used to realized a NOT- gate (solid blue line). The dashed lines represent the separatrices. The black arrows indicate the direction of the dynamics of the classical angular momentum.

is equal to $2\arccos k$. We thus need to apply a $2\arccos k$- pulse along $\vec{e}_3$ when the Bloch vector belongs to the equator. Note that in the adiabatic limit, which corresponds here to $k \to 1$, this angle tends to zero. In the latter case, the method is equivalent to a 180° BIR-1 pulse [27], with an Allen-Eberly hyperbolic shape. The details of the pulse are given in Tab. VII. The robustness of the control process is shown in Fig. 16 for a control field of the

$t \in \left[0, \frac{T}{2} - \frac{T_\Delta}{2}\right]$	$t \in \left[\frac{T}{2} - \frac{T_\Delta}{2}, \frac{T}{2} + \frac{T_\Delta}{2}\right]$	$t \in \left[\frac{T}{2} + \frac{T_\Delta}{2}, T\right]$
$\Omega = \frac{1}{\sqrt{1-k^2}}\mathrm{sech}(t+\rho)$<br>$\Delta = -\frac{k}{\sqrt{1-k^2}}\tanh(t+\rho)$	$\Omega = 0$<br>$\Delta = 2\frac{\arccos(k)}{T_\Delta}$	$\Omega = -\frac{1}{\sqrt{1-k^2}}\mathrm{sech}(t+\rho)$<br>$\Delta = \frac{k}{\sqrt{1-k^2}}\tanh(t+\rho)$
$\rho = -\frac{T-T_\Delta}{2}$		$\rho = -\frac{T+T_\Delta}{2}$

TABLE VII. Details of the pulse used to implement robust- NOT gates. T is the total duration of the process and T_Δ is the duration of the constant pulse along $\vec{e}_3$.

form:

$$\Omega^{(\alpha)} = (1+\alpha)\Omega, \Delta^{(\alpha)} = \Delta, \tag{55}$$

where α is a scaling factor corresponding to the fields inhomogeneities. The fidelity J is defined such that $J = \text{Trace}({}^t R(t_f) \cdot R_f)$, where R_f is the target rotation matrix given by:

$$R_f = \begin{pmatrix} -1 & 0 & 0 \\ 0 & 1 & 0 \\ 0 & 0 & -1 \end{pmatrix}. \tag{56}$$

The fidelity has been also computed for a standard π- pulse and for the TRE- pulse adapted to configuration (a) in order to compare the three methods.

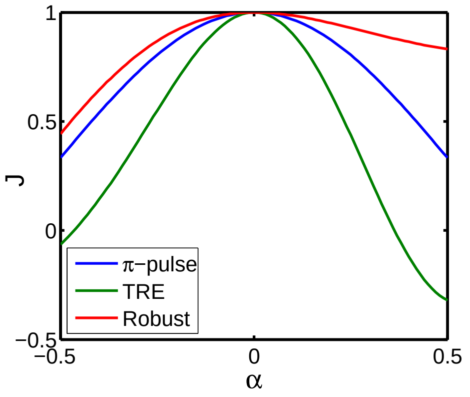

FIG. 16. (Color online) Robustness of the NOT gate implemented with a standard π- pulse (blue or black), a TRE- pulse (green or light gray), and with the method detailed above (red of dark gray), for $k = 0.9$. The robustness of the method can be improved by choosing k closer to 1.

VI. NUMERICAL IMPLEMENTATION OF THE TRAJECTORIES OF A RIGID BODY AND OF A CONTROL PULSE SEQUENCE

We provide in this supplementary material a Matlab code to compute the trajectories of a rigid body as a function of the parameters k and E. We also deduce the associated control fields and the dynamics of the Bloch vector for a state to state transfer. This code can be used by the interested reader to test some of the control protocols proposed in this work.

VII. JACOBI'S ELLIPTIC FUNCTIONS

We recall in this paragraph some standard properties of Jacobi's elliptic functions used in this paper [10]. Jacobi's elliptic functions generalize the standard trigonometric functions.

This is a family of functions which includes cosine, sine and hyperbolic functions. They are written as follows: $\mathrm{sn}(u,m)$, $\mathrm{cn}(u,m)$ and $\mathrm{dn}(u,m)$. Their periods are related to the complete elliptic integral of the first kind $\mathrm{K}(m)$ as $4\mathrm{K}(m)$, $4\mathrm{K}(m)$ and $2\mathrm{K}(m)$, respectively, with m a parameter belonging to the interval $[0,1]$. The elliptic integral is defined as:

$$\mathrm{K}(m) = \int_0^1 \frac{dx}{\sqrt{1-x^2}\sqrt{1-mx^2}}.$$

In the case $m=0$, we have $\mathrm{K}(m)=\pi/2$ and the functions $\mathrm{sn}(u,m)$, $\mathrm{cn}(u,m)$ and $\mathrm{dn}(u,m)$ become $\sin(u)$, $\cos(u)$ and 1, respectively. In the case $m=1$, we obtain $K(m)=+\infty$ and the elliptic functions can be identified with $\tanh(u)$, $\mathrm{sech}(u)$ and $\mathrm{sech}(u)$. For a value of m such that $m=1-\varepsilon$ with ε a small positive parameter, the function $\mathrm{K}(m)$ is, to the first order, given by:

$$\mathrm{K}(1-\epsilon) = \frac{1}{2}\ln\left(\frac{8}{\varepsilon}\right) + O(\varepsilon). \qquad (57)$$

Figure 17 shows Jacobi's elliptic functions for $m=1/2$.

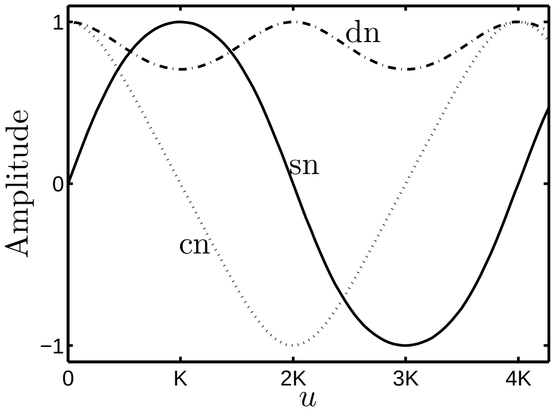

u	0	$\mathrm{K}(m)$	$2\mathrm{K}(m)$	$3\mathrm{K}(m)$
$\mathrm{sn}(u,m)$	0	1	0	-1
$\mathrm{cn}(u,m)$	1	0	-1	0
$\mathrm{dn}(u,m)$	1	$\sqrt{1-m}$	1	$\sqrt{1-m}$

FIG. 17. (Color online) Plot of Jacobi's Elliptic functions in terms of u for $m=1/2$. The Table gives some particular values of the same functions.

These functions satisfy the following relations:

$$\mathrm{sn}^2(u,m) + \mathrm{cn}^2(u,m) = 1,$$
$$m\,\mathrm{sn}^2(u,m) + \mathrm{dn}^2(u,m) = 1.$$

Primitive	Function	Derivative
$\frac{1}{\sqrt{m}}\ln\Big(\mathrm{dn}(u,m)-\sqrt{m}\,\mathrm{cn}(u,m)\Big)$	$\mathrm{sn}(u,m)$	$\mathrm{cn}(u,m)\mathrm{dn}(u,m)$
$\frac{1}{\sqrt{m}}\arctan\left(\frac{\sqrt{m}\,\mathrm{sn}(u,m)}{\mathrm{dn}(u,m)}\right)$	$\mathrm{cn}(u,m)$	$-\mathrm{sn}(u,m)\mathrm{dn}(u,m)$
$\mathrm{am}(u,m)$	$\mathrm{dn}(u,m)$	$-m\mathrm{sn}(u,m)\mathrm{cn}(u,m)$

TABLE VIII. Derivatives and primitives of the Jacobi's elliptic functions.

The derivatives and the primitives of sn, cn and dn are given in Tab. VIII.

---

[1] L. D. Landau and E. M. Lifshitz, *Mechanics* (Pergamon Press, Oxford, 1960).

[2] H. Goldstein, *Classical Mechanics* (Addison-Wesley, Reading M. A., 1950).

[3] M. H. Levitt, *Spin Dynamics: Basics of Nuclear Magnetic Resonance* (Wiley, New York, 2008).

[4] R. R. Ernst, G. Bodenhausen and A. Wokaun, *Principles of Nuclear Magnetic Resonance in One and Two Dimensions* (Oxford Science Publications, 2004).

[5] D. Daems, A. Ruschhaupt, D. Sugny and S. Gurin, *Robust Control by a Single-Shot Shaped Pulse*, Phys. Rev. Lett. **111**, 050404 (2013)

[6] L. Allen and J. H. Eberly, *Optical Resonance and Two-Level Atoms*, (Wiley, New York, 1975).

[7] L. Van Damme, P. Pavao Mardešić and D. Sugny, *The tennis racket effect in a three-dimensional rigid body*, Physica D **338**, 17 (2017)

[8] M. S. Ashbaugh, C. C. Chiconc and R. H. Cushman, *The twisting tennis racket*, J. Dyn. Diff. Eq. **3**, 67 (1991)

[9] N. V. Vitanov, T. Halfman, B. W. Shore and K. Bergmann, *Laser-induced population transfer by adiabatic passage techniques*, Annu. Rev. Phys. Chem. **52**, 763 (2001)

[10] M. Abramowitz, *Handbook of Mathematical Functions*, (Dover Publications, New York, 1964).

[11] K.-A. Suominen and B. M. Garraway, *Population transfer in a level-crossing model with two time scales*, Phys. Rev. A **45**, 1 (1992)

[12] F. T. Hioe and C. E. Carroll, *Two-state problems involving arbitrary amplitude and frequency modulations*, Phys. Rev. A **32**, 1541 (1985)

[13] F. T. Hioe, *Solution of Bloch equations involving amplitude and frequency modulations*, Phys.

Rev. A **30**, 2100 (1984)

[14] B. Bonnard and D. Sugny, *Optimal control with applications in space and quantum dynamics*, AIMS: Applied mathematics Vol. 5 (2012).

[15] R. Montgomery, *How much does the body rotate? A Berry's phase from the 18th century*, Am. J. Phys. **59**, 394 (1991)

[16] J. Natário, *An elementary derivation of the Montgomery phase formula for the Euler top*, J. Geom. Mech. **2**, 113 (2010)

[17] A. Bohm, A. Mostafazadeh, H. Koizumi, Q. Niu and J. Zwanziger, *The geometric phase in quantum systems*, Springer (2003)

[18] M. V. Berry, *Quantal phase factors accompanying adiabatic changes*, Proc. Roy. Soc. London, Ser. A **392**, 45 (1984)

[19] Y. Aharanov and J. Anandan, *Phase change during a cyclic quantum evolution*, Phys. Rev. Lett. **58**, 1593 (1987)

[20] E. Sjöqvist, *A new phase in quantum computation*, Physics, **1**, 35 (2008)

[21] A. Ekert, M. Ericsson, P. Hayden, H. Inamori, J. A. Jones, D. K. L. Oi and V. Vedral, *Geometric quantum computation*, J. Mod. Opt. **47**, 2501 (2000)

[22] J. A. Jones, V. Vedral, A. Eckert and G. Castagnoli, *Geometric quantum computation with NMR*, Nature **403**, 869 (2000).

[23] W. Xiand-Bin and M. Keiji, *Nonadiabatic conditional geometric phase shift with NMR*, Phys. Rev. Lett. **87**, 097901 (2001)

[24] S.-L. Zhu and Z. D. Wang, *Implementation of univeral quantum gates based on nonadiabatic geometric phases*, Phys. Rev. Lett. **89**, 097902 (2002)

[25] Y. Ota, Y. Goto, Y. Kondo and M. Nakahara, *Geometric quantum gates in liquid state NMR based on a cancellation of dynamical phases*, Phys. Rev. A **80**, 052311 (2009)

[26] S. L. Zhu and Z. D. Wang, *Universal quantum gates based on a pair of orthogonal cyclic states: Application to NMR systems*, Phys. Rev. A **67**, 022319 (2003)

[27] A. Tannús and M. Garwood, *Adiabatic pulses*, NMR Biomed **10**, 423 (1997)

[28] M. Garwood and Y. Ke, *Symmetric pulses to induce arbitrary flip angles with compensation for RF inhomogeneity and resonance offsets*, J. Magn. Reson. **94**, 511 (1991)

```
clear all
close all
clc
%%%%%%%%%%%%%%%%%%%%%%%%%%%%%%%%%%%%%%%%%%%%%%%%%%%%%%%%%%%%%%%%%%
%Choose a set of parameters (k,E)
k=0.7;
E=0.25;
%The separatrix is given for E=k^2/2
%%%%%%%%%%%%%%%%%%%%%%%%%%%%%%%%%%%%%%%%%%%%%%%%%%%%%%%%%%%%%%%%%%
%Compute the parameters of elliptic functions
if 2*E < k^2      %Oscillating
    w   = k*sqrt(1-2*E);
    m   = 2*E*(1-k^2)/(k^2*(1-2*E)); %Ellipticity
    rho = ellipke(m);                %Initial phase
    tf  = 2*rho;                     %Final time=1/2 period
elseif 2*E > k^2  %Rotating
    w   = sqrt(2*E*(1-k^2));
    m   = k^2*(1-2*E)/(2*E*(1-k^2));
    rho = ellipke(m);
    tf  = 2*rho;
elseif 2*E == k^2 %Separatrix
    w   = k*sqrt(1-k^2);
    rho = -10;
    tf  = 2*abs(rho);
end
%Define the numerical control time
N=1000;
t=linspace(0,tf,N);
%%%%%%%%%%%%%%%%%%%%%%%%%%%%%%%%%%%%%%%%%%%%%%%%%%%%%%%%%%%%%%%%%%
%Control fields and angular momentum
if 2*E < k^2      %Oscillating trajectory
    %Compute the elliptic function cn sn dn
    [sn cn dn]=ellipj(t+rho,m);
    %Control fields
    w1 = (-sqrt(2*E)/w)*cn;
    w2 = (k*sqrt(2*E)/w)*sn;
    %Angular momentum
    L1 = (-sqrt(2*E))*cn;
    L2 = (sqrt(2*E)/k)*sn;
    L3 = (w/k)*dn;
elseif 2*E > k^2  %Rotating trajectory
    %Compute the elliptic function cn sn dn
    [sn cn dn]=ellipj(t+rho,m);
    %Control fields
    w1 = (sqrt(2*E)/w)*dn;
    w2 = (k*sqrt(2*E)*sqrt(m)/w)*sn;
    %Angular momentum
    L1 = (sqrt(2*E))*dn;
    L2 = (sqrt(2*m*E)/k)*sn;
    L3 = (-w*sqrt(m)/k)*cn;
elseif 2*E == k^2 %Separatrix
    %Control fields
    w1 = (k/w)*sech(t+rho);
```

```
    w2 = (k^2/w)*tanh(t+rho);
    %Angular momentum
    L1 = k*sech(t+rho);
    L2 = tanh(t+rho);
    L3 = (w/k)*sech(t+rho);
end
%%%%%%%%%%%%%%%%%%%%%%%%%%%%%%%%%%%%%%%%%%%%%%%%%%%%%%%%%%%%%%%%%%
%Integration of the Bloch equation
%Basis
Sx=[0 0 0;0 0 -1;0 1 0];
Sy=[0 0 1;0 0 0;-1 0 0];
%State tabular
M=zeros(3,N+1);
M(:,1)=[0;1;0]; %Example of initial state: y-axis of the Bloch sphere
dt=t(2)-t(1);
%Propagation
for n=1:N
    H=w1(n)*Sx+w2(n)*Sy; %Propagator
    M(:,n+1)=expm(H*dt)*M(:,n);
end
M1=M(1,:);
M2=M(2,:);
M3=M(3,:);
tM=linspace(0,tf,N+1); %Time of length N+1
%%%%%%%%%%%%%%%%%%%%%%%%%%%%%%%%%%%%%%%%%%%%%%%%%%%%%%%%%%%%%%%%%%
%%%%%%%%%%%%%%%%%%%%%%%%%%%%%%%%%%%%%%%%%%%%%%%%%%%%%%%%%%%%%%%%%%
figure(1) % plot of the two control fields
plot(t,w1,'-b',t,w2,'-r','linewidth',2);
xlabel('time','Fontsize',18)
ylabel('\omega_1, \omega_2','Fontsize',18)
%%%%%%%%%%%%%%%%%%%%%%%%%%%%%%%%%%%%%%%
figure(2) % plot of the time evolution of the Bloch vector
plot(tM,M1,'-b',tM,M2,'-r',tM,M3,'-k','linewidth',2);
xlabel('time','Fontsize',18)
ylabel('M_1, M_2, M_3','Fontsize',18)
set(gca,'YTick',-1:0.2:1,'Fontsize',12)
axis([0,max(t),-1,1]);
%%%%%%%%%%%%%%%%%%%%%%%%%%%%%%%%%%%%%%%
figure(3); % plot of the dynamics of the angular momentum and of the Bloch vector
% on the Bloch sphere.
hold on;
plot3(L1,L2,L3,'-k','linewidth',2);
plot3(M1,M2,M3,'--k','linewidth',2);
h=legend('$\vec{L}$','$\vec{M}$');
set(h,'interpreter','latex','fontsize',16)
[xs ys zs]=sphere(100);
surfl(xs,ys,zs)
shading interp
colormap gray
alpha 0.7
axis square
grid on
```

```
view(147,34)
set(gca,'XTick',-1:0.5:1,'Fontsize',12)
set(gca,'YTick',-1:0.5:1,'Fontsize',12)
set(gca,'ZTick',-1:0.5:1,'Fontsize',12)
xlabel('e_1')
ylabel('e_2')
zlabel('e_3')
%%%%%%%%%%%%%%%%%%%%%%%%%%%%%%%%%%%%%%%%%%%%%%%%%%%%%%%%%%%%%
```
